# The Value of Circulating Circular RNA in Cancer Diagnosis, Monitoring, Prognosis, and Guiding Treatment

**DOI:** 10.3389/fonc.2021.736546

**Published:** 2021-10-14

**Authors:** Yunjing Zhang, Ying Wang, Xinwan Su, Ping Wang, Weiqiang Lin

**Affiliations:** ^1^ Department of Nephrology, The Fourth Affiliated Hospital, and Institute of Translational Medicine, Zhejiang University School of Medicine, Jinhua, China; ^2^ Department of Urology, The First Affiliated Hospital, Zhejiang University School of Medicine, Hangzhou, China

**Keywords:** cancer diagnosis, liquid biopsy, circRNA, biomarker, exosome

## Abstract

Liquid biopsy includes non-invasive analysis of circulating tumor-derived substances. It is a novel, innovative cancer screening tool that overcomes the limitations of current invasive tissue examinations in precision oncology. Circular RNA (circRNA) is a recent, novel, and attractive liquid biomarker showing stability, abundance, and high specificity in various diseases, especially in human cancers. This review focused on the emerging potential of human circRNA in body fluids as the liquid biopsy biomarkers for cancers and the methods used to detect the circRNA expression and summarized the construction of circRNA biomarkers in body fluids for treating human cancers and their limitations before they become part of routine clinical medicine. Furthermore, the future opportunities and challenges of translating circRNAs in liquid biopsy into clinical practices were explored.

## Introduction

Cancer is one of the leading causes of death worldwide, with more than 10 million people deaths estimated in 2020, and the morbidity and mortality rates are increasing. The development of “omics” technology has facilitated the progress of precision oncology, which includes tailoring treatment plans based on the molecular characteristics of each patient’s tumor (1). In the current era of precision oncology, liquid biopsy specimen collection is far less invasive than tissue specimen collection and can be repeated several times during the follow-up process to achieve real-time monitoring. Liquid biopsy can eliminate the tumor heterogeneity effect by determining the genomic profile of cancer patients, assist with a systemic and comprehensive representation of disease, help with early cancer detection (even before radiological and imaging tests detect the presence of tumors), aid in evaluating therapeutic reaction, detect minimal residual disease, and test for cancer cells’ drug resistance. Besides blood, several other body fluids such as urine (2), saliva (3), pleural effusions (4), and cerebrospinal fluid (CSF) (5) as well as stool (6) can be used to reveal information about the tumor. Biofluid sampling and analysis provide an accurate account of the molecular traits of primary tumors and distant metastases. Most liquid biopsy analyses have displayed their clinical utility in human cancers (7–10). The first important milestone in this field was reached in 2016 with the Food and Drug Administration (FDA) approval of the first companion diagnostic test for lung cancer based on the circulating tumor DNA (ctDNA) content of a liquid biopsy. Besides ctDNA, several other biomarkers in circulating body fluids have been studied, such as circulating tumor cells (CTCs) (11), extracellular vesicles (EVs) (12), cell-free RNA (cfRNA) (13), circulating proteins (14), circulating metabolites (15), and platelets (16). Among these, RNA-based liquid biomarkers have received increasing attention because of their varied expression levels and strong links with cancer (17).

CircRNA stems from spliceosome-mediated non-sequential backsplicing of precursor mRNA (pre-mRNA); that is, the downstream 5′ splice donor site is connected to the upstream 3′ splice acceptor site (18). They were found in RNA viruses as viroids for the first time in 1976 and have been considered a result of splicing errors for several years (19). CircRNAs can be divided into five categories: exonic circular RNAs (ecircRNAs) (20, 21), intronic circular RNAs (ciRNAs) (20, 22), exon–intron circular RNAs (EIciRNAs) (21), intergenic circRNAs (20), and antisense circular circRNAs (20). EcircRNA, mostly distributed in the cytoplasm, accounts for nearly 85% of all recognized circRNAs (20). CiRNAs and EIciRNAs are primarily concentrated in the nucleus, indicating that they may have different biological functions (23).

**Figure 1:**
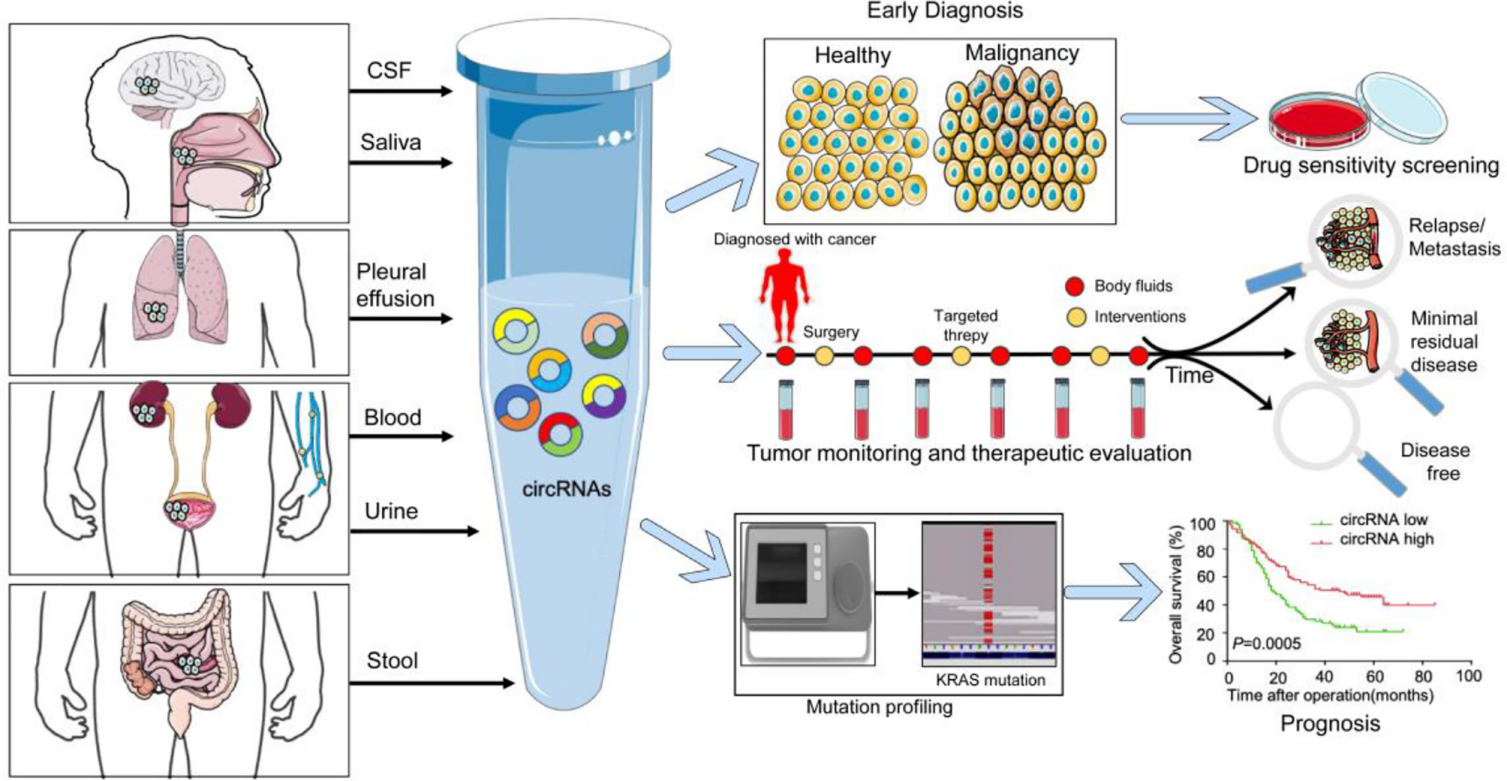
The value of circRNA as a liquid biopsy marker in tumor diagnosis and precision medicine. Schematic representation of different body fluids that contain circRNAs, specifically including cerebrospinal fluid (CSF), saliva, pleural effusions, blood, urine, and stool. The localization of the primary or metastatic lesions influences the presence of circulating circRNAs in individual body fluids. As indicated, cancers can be detected through analysis of the circRNA content derived from the biofluids and further predict the sensitivity of treatment drugs. Longitudinal monitoring of circRNAs could be used to monitor tumor progression and therapeutic effects. Genomic mutation associated with the circRNA expression could be used to assess prognosis.

Compared to the traditional tumor tissue biopsy biomarkers, circRNAs are novel, convenient, and non-invasive liquid biopsy biomarkers. Their high abundance and stability (24), abundant expression (25), and high specificity (26) make circRNAs a promising biomarker for various diseases, particularly cancers (27). Lots of research have been done to study circRNAs as a liquid biopsy biomarker in cancer (**Figure 1**) owing to the advantages they offer (27) and the significance of liquid biopsy biomarkers in precision medicine (28). In this review, we have summarized the latest information about circRNAs in liquid biopsy, including their biological significance in the development and progression of cancer, their potency in cancer diagnosis, monitoring, and prognosis and outlined the advantages and limitations of available methods used to detect the circRNA expression and their different roles in various human cancers. Moreover, the various circRNA biomarkers constructed for treating human tumors were summarized. We have further investigated future opportunities and challenges of translating circRNAs into clinical applications and outlined significant technological advances that may overcome these shortcomings.

## Biological Function of Body-Fluid CircRNAs in Cancer

The potential functions of circRNAs have been extensively studied. It can regulate fundamental biological processes and display an essential function in various human illnesses, including cancers (29–34). One of the most important functions of circRNAs is their role in cell viability and distant metastasis.

### CircRNAs and Proliferation

Proliferation disorder is one of the critical elements of tumor transformation, making the cell-cycle regulation mechanism a much sought-after research topic (35). Xue et al. demonstrated that exosomal circRNA_100284 acts as the microRNA-217 (miR-217) sponge (36), regulating the cell cycle by inducing G2/M phase arrest, inhibiting cell proliferation, and targeting EZH2 (proliferation indicator) in various cancers (37, 38). Exosomal circSATB2 promotes the cell proliferation of non-small lung cancer cells (NSLCCs) *via* sponging miR-326 (39). These proofs validate the role of exosomal circRNAs in regulating the cell cycle and cell proliferation. Another research reported a significantly higher circRASSF2 level of laryngeal squamous cell carcinoma (LSCC) in serum exosomes than the normal groups (40). Moreover, knockdown of circ-RASSF2 remarkably suppressed cell proliferation through the miR-302b-3p/insulin-like growth factor 1 (IGF-1R) axis, suggesting the importance of serum circRNAs in tumor proliferation.

### CircRNAs and Metastasis

The increasing evidence shows that the circRNA expression in certain biofluids is associated with cancer progression and metastasis. For instance, Lu et al. demonstrated a critical role of plasma circ-RanGAP1 in gastric cancer (GC). They showed that the expression level of circ-RanGAP1 in GC and plasma was closely connected with the advanced tumor-node-metastasis (TNM) stage, lymph node metastases, and low survival rate. Circ-RanGAP1 has been shown to accelerate tumor-cell growth by mediating the miR-877-3a/vascular endothelial growth factor A (VEGFA axis) (41). Likewise, a recent study verified that enriched circ-133 in the plasma exosome of colorectal cancer cells (CRC) patients could promote cancer metastasis *via* circ-133-mediated miR-133a/guanine nucleotide exchange factor-H1 (GEF-H1)/Ras homolog family member A (RhoA) signaling pathway, suggesting that exosomal circ-133 can be a surveillance biomarker for progression of CRC (42). This phenomenon has also been observed in hepatocellular carcinoma cells and patients (43). Chen et al. have also reported significantly increased circPRMT5 expression in serum and urine of patients with urothelial carcinoma of the bladder (UCB) (44). The research also reported that exosomal circPRMT5 facilitated the epithelial–mesenchymal transition (EMT) of UCB cells through miR-30c/SNAIL1/E-cadherin signaling.

## CircRNAs in Body Fluids Are Promising Biomarkers for Cancer

A good clinically significant biomarker must fit the analytical and clinical validity and clinical utility standards (45). As a newly identified noncoding RNA, circRNA has obvious advantages over typical linear RNAs as an illness biomarker (27). The circular covalent bond structure of circRNAs makes them highly resistant to exonuclease degradation and prolongs their lifespan (24, 46). Additionally, circRNAs express themselves abundantly in various human tissue samples, such as saliva (47) and plasma (48), and even in serum exosomes (49), indicating their role as suitable biomarkers for liquid biopsy (**Figure 1**). A number of circRNAs have been considered as biomarkers of human diseases recently due to the high stability of circRNAs in mammalian cells (27), especially in some malignancies (50–53). A study with 1,195 plasma samples used plasma circRNAs to diagnose hepatitis B virus (HBV)-related hepatocellular carcinoma (HCC) (54). The study reported that the plasma expression levels of hsa_circ_0000976, hsa_circ_00077550, and hsa_circ_0139897 in HCC patients are significantly higher than those in the control group. Lin et al. (55) also found that the plasma levels of circ-CCDC66, circ-ABCC1, and circ-STIL are significantly reduced in CRC patients compared to the control group. The sensitivity and specificity of the combined diagnosis of CRC using these three circRNAs were 64.4% and 85.2%, respectively. Gastric cancer is the most widely studied category of cancer in the field of circRNA research, and some publications have depicted the dysregulation of circRNAs in plasma such as hsa_circ_0001017, hsa_circ_0061276 (56), hsa_circ_0000745 (57), hsa_circ_0000520 (58), hsa_circ_0000190 (59), hsa_circ_0001649 (60), hsa_circ_0000181 (61), and circ_002059 (50), all of which have displayed diagnostic effectiveness. A markedly downregulated expression of hsa_circ_002059 was detected in GC tissues compared to the matched para-carcinoma tissues (50). Different expressions of hsa_circ_002059 in plasma were detected in 36 paired plasma samples from pre- and postoperative GC patients. A low expression of hsa_circ_002059 was closely associated with age, gender, TNM stage, and distant metastasis. The assessment of the diagnostic value of hsa_circ_002059 showed that its sensitivity and specificity were 81.0% and 62.0%, respectively, and the area under the curve (AUC) was 0.73, suggesting hsa_circ_002059 to be a potentially stable GC biomarker. Tan et al. reported a fusion circRNA called F-circEA, derived from an EML4-ALK fusion gene, and it is positively expressed in five of six NSCLC patients with EML4-ALK translocation (62). F-circEA also exists in the plasma of EMLA4-ALK-positive NSCLC patients specifically.

CircRNAs have broad application prospects in monitoring the treatment efficacy and assessing cancer prognosis. Li et al. identified more than 1,000 circRNAs in human serum exosomes for the first time and proposed that these circRNAs could distinguish colon cancer patients from the healthy controls (46). The decreased expression of circKIAA1244 in plasma (63) and increased expression of hsa_circ_0000467 (64) were closely correlated with the shorter overall survival time of GC patients. Pan et al. (65) used quantitative reverse transcriptase-polymerase chain reaction (qRT-PCR) to detect the expression of hsa-circ-0004771 in circulating exosomes of 170 patients and 45 healthy controls and pointed out that the hsa-circ-0004771 expression in the serum of CRC patients was significantly upregulated compared to patients with benign enteral illness and hematopoietic cytokines (HCs). The hsa-circ-0004771 expression level in CRC patients was 14 times higher than that of HCs, and it was closely related to stage and distal metastasis. The sensitivity and specificity of hsa-circ-0004771 to differentiate CRC patients from HCs were 80.91% and 82.86%, respectively. These results indicated that the exosomal hsa-circ-0004771 overexpression was the source of tumors and can be used as biomarkers for CRC diagnosis. The expression level of circ_0000285 in sera of 150 patients with nasopharyngeal carcinoma was elevated compared to a healthy control; this level was correlated with the tumor size, degree of differentiation, lymph node metastasis, distal metastasis, and the TNM stage (66). Moreover, the expression level increased by more than three times in radiotherapy-resistant patients. Li et al. (67) detected high expression of circ-PDE8A in liver metastatic pancreatic ductal adenocarcinoma (PDAC) tissues, confirming that this feature is closely related to lymphatic infiltration and TNM stage. Circ-PDE8A is an independent risk factor affecting the survival of PDAC patients; further study verified the presence of circ-PDE8A-rich exosomes secreted by tumor cells in the plasma of PDAC patients. Similarly, circ-PDE8A in plasma exosomes may also be a diagnostic and prognostic marker of PDAC.

In addition to plasma, serum, and exosome, circRNAs were also found in some other body fluids such as saliva (47, 68, 69), gastric juice (70), and urine (44). Saliva is a body fluid used in disease research due to its convenient and non-invasive sampling method. When compared with healthy controls, the saliva of oral squamous cell carcinoma (OSCC) patients showed increased levels of hsa_circ_0001874 and hsa_circ_0001971 (69). Clinical data results associated salivary hsa_circ_0001874 with TNM stage and tumor grade, and hsa_circ_0001971 with TNM stage. Taken together, hsa_circ_0001874 and hsa_circ_0001971 display the AUC value to be 0.922. These results revealed the possibility of salivary hsa_circ_0001874 and hsa_circ_0001971 as the OSCC diagnostic biomarkers. Bahn et al. (47) detected and confirmed the presence of circRNAs in cell-free saliva, providing a new direction for using circRNAs as biomarkers. Gastric juice examination is a highly organ-specific test for the diagnosis of gastric diseases. Shao et al. discussed the feasibility of hsa_circ_0014717 in gastric juice as a biomarker for screening high-risk GC patients (70). The expression of hsa_circ_0014717 in gastric juice of 38 healthy individuals, 30 gastric ulcer patients, 15 chronic atrophic gastritis patients, and 39 GC patients was detected by qRT-PCR. Hsa_circ_0014717 was significantly downregulated in patients with chronic atrophic gastritis than the healthy control, indicating that hsa_circ_0014717 in gastric juice can be used as a biomarker for screening high-risk GC groups. Additionally, Chen et al. detected high expression of circPRMT5 in exosomes isolated from the urine samples of patients with UCB (44). Additional studies are listed in **Table 1**.

**Table 1:** Studies of circRNA as liquid biopsy cancer biomarkers.

Disease	CircRNA biomarker	Expression change	Biofluids	Significance	Cancer *vs.* control	Ref.
GC	hsa_circ_0000190	Down	Plasma	Diagnostic	140 *vs.* 140	(59)
	hsa_circ_002059	Up	Plasma	Diagnostic	36 *vs.* 36	(71)
	hsa_circ_0000745	Down	Plasma	Diagnostic	60 *vs.* 60	(57)
	hsa_circ_0001017	Down	Plasma	Diagnostic	121 *vs.* 121	(56)
	hsa_circ_0061276	Down	Plasma	Prognostic		
	circKIAA1224	Down	Plasma	Diagnostic	62 *vs.* 25	(63)
	hsa_circ_0000520	Down	Plasma	Diagnostic	45 *vs.* 17	(58)
	hsa_circ_0014717	Down	Gastric juice	Diagnostic	39 *vs.* 38	(70)
	hsa_circ_0001821	Down	Whole blood	Diagnostic	30 *vs.* 30	(72)
	circ-RanGAP1	Up	Exosome	Prognostic	30 *vs.* 30	(41)
	hsa_circ_0001649	Down	Serum	Diagnostic and monitoring	20 *vs.* 20	(60)
	hsa_circ_0000467	Up	Plasma	Diagnostic and prognostic	20 *vs.* 20	(64)
	hsa_circ_0000181	Down	Plasma	Diagnostic	102 *vs.* 105	(61)
	circNRIP1	Up	Exosome	Diagnostic	40 *vs.* 40	(73)
LAC	hsa_circ_0013958	Up	Plasma	Diagnostic	30 *vs.* 30	(74)
	hsa_circ_0000190	Up	Plasma	Diagnostic and monitoring	231 *vs.* 41	(75)
	circMAN1A2	Up	Serum	Diagnostic	45 *vs.* 121	(76)
NSCLC	hsa_circ_0102533	Up	Whole blood	Diagnostic	41 *vs.* 26	(77)
	circFARSA		Plasma	Diagnostic	50 *vs.* 50	(78)
SCLC	circFECR1	Up	Exosome	Prognostic	61 *vs.* 55	(79)
PC	circLDLRAD3	Up	Plasma	Diagnostic	31 *vs.* 31	(80)
HCC	circ_0001445	Down	Plasma	Diagnostic	104 *vs.* 153	(81)
	circPTGR1	Up	Exosome	Prognostic	82 *vs.* 47	(82)
	hsa_circ_0000798	Up	PBMC	Prognostic	72 *vs.* 30	(83)
	hsa_circ_100338	Up	Exosome	Diagnostic and prognostic	16 *vs.* 23	(84)
	circ-ZNF652	Up	Exosome	Prognostic	33 *vs.* 33	(85)
BC	hsa_circ_0001785	Up	Peripheral blood	Prognostic	5 *vs.*5	(86)
UCB	circPRMT5	Up	Urine	Diagnostic	71 *vs.* 36	(44)
			Exosome	Prognostic		
			Serum	Diagnostic	18 *vs.* 14	
			Exosome	Prognostic		
CRC	circFMN2	Up	Serum	Prognostic	35 *vs.* 27	(87)
	circIFT80	Up	Plasma	Prognostic	5 *vs.* 5	(88)
	hsa_circ_0001649	Up	Serum	Diagnostic	9 *vs.* 9	(60)
	hsa-circ-0000338	Up	Serum	Predictive	10 *vs.* 7	(89)
	hsa-circ-0004771	Up	Serum	Diagnostic and prognostic	135 *vs.* 45	(65)
	circ-KLDHC10	Up	Serum	Diagnostic	11 *vs.* 11	(46)
	circVAPA	Up	Plasma	Diagnostic	60 *vs.* 43	(90)
NPC	hsa_circ_0000285	Up	Serum	Diagnostic and prognostic	150 *vs.* 100	(66)
Oral cancer	circMAN1A2	Up	Serum	Diagnostic	100 *vs.* 121	(76)
	hsa_circ_0001874	Up	Saliva	Diagnostic	93 *vs.* 85	(69)
	hsa_circ_0001971	Up	Saliva	Diagnostic	93 *vs.* 85	(69)
CSCC	hsa_circ_0101996	Up	Whole blood	Diagnostic	87 *vs.* 55	(91)
	hsa_circ_0101119	Up	Whole blood	Diagnostic	87 *vs.* 55	(91)
AML	hsa_circ_0004277	Down	Mononuclear cell	Diagnostic	115 *vs.* 12	(92)
CML	hsa_circ_100053	Up	PBMC	Prognostic	150 *vs.* 100	(93)
Thyroid cancer	circMAN1A2	Up	Serum	Diagnostic	57 *vs.* 121	(76)
	hsacirc_007293	Up	Serum Exosome	Diagnostic	122 *vs.* 163	(94)
	hsacirc_020135	Up	Serum Exosome	Diagnostic	122 *vs.* 163	
	hsacirc_031752	Up	Serum Exosome	Prognostic	122 *vs.* 163	
Ovarian cancer	circMAN1A2	Up	Serum	Diagnostic	36 *vs.* 36	(76)
Osteosarcoma	hsa_circ_0000885	Up	Serum	Diagnostic	25 *vs.* 25	(95)
Endometrial cancer	hsa_circ_0109046	Up	Serum Exosome	Diagnostic	10 *vs.* 10	(96)
	hsa_circ_0002577	Up	Serum Exosome	Diagnostic	10 *vs.* 10	(96)
Prostate cancer	hsa_circ_0044516	Up	Exosome	Diagnostic	6 *vs.* 6	(97)

GC, gastric cancer; LAC, lung adenocarcinoma; NSCLC, non-small cell lung cancer; SCLC, small cell lung cancer; PC, pancreatic cancer; HCC, hepatocellular carcinoma; BC, breast cancer; UCB, urothelial carcinoma of the bladder; CRC, colorectal cancer; NPC, nasopharyngeal carcinoma; CSCC, cervical squamous cell carcinoma;AML, acute myeloid leukemia; CML, chronic myeloid leukemia.

CircRNAs have also been used to evaluate the effectiveness of tumor-treatment methods and supervise tumor stress. For instance, Xie et al. (70) demonstrated an abundant expression of circSHKBP1 (hsa_circ_0000936) in GC serum and linked the abundance with the late TNM stage and low survival rates. The exosomal circSHKBP1 level metastasized early after gastrectomy was significantly reduced. Notably, the expression of circRNA reflects the clinical dynamics of sequential therapy and might offer a promising scheme for mirroring tumor stress and prognosis. The hsa_circ_0001649 expression levels in matched serum samples of patients with GC before and after surgery indicated that serum hsa_circ_0001649 expression levels in GC patients increase significantly after surgery. Also, the hsa_circ_0001649 expression in poorly differentiated and undifferentiated tumors was significantly lower than that in the well-differentiated tumors, indicating a negative correlation with the pathological differentiation of GC (60). The sensitivity and specificity of the receiver operating characteristic (ROC) curve were 0.711 and 0.816, respectively, and the AUC was 0.834. Therefore, hsa_circ_0001649 can be used as a blood-based biomarker to supervise illness progression and cure response. Ping et al. also found that compared to healthy people, circ_100053 expression in serum of patients with chronic myeloma leukemia (CML) was significantly upregulated. Additionally, the circ_100053 level in imatinib-resistant patients was almost the same as that of imatinib-sensitive patients, whereas it was four times in CML patients. Therefore, high circ_100053 expression predicts a poor prognosis and imatinib resistance in CML patients (93). Furthermore, circ-SORE mediated sorafenib resistance in HCC partially through its combination with YBX1 (98). There are also reports that hsa_circ_0005963 derived from extracellular vesicles induces CRC resistance through the miR-122-PKM2 pathway (99). Another study reported that has_circ_0035483 was highly expressed in RCC and promoted gemcitabine resistance by regulating the hsa-miR-355/CCNB1 axis. Reducing the expression of has_circ_0035483 can enhance gemcitabine sensitivity (100). A confirmed relationship between circRNA expression and drug sensitivity would guide the rational clinical use of drugs and improve patients’ prognosis.

## Detection of CircRNAs in Body Fluids

**Figure 2:**
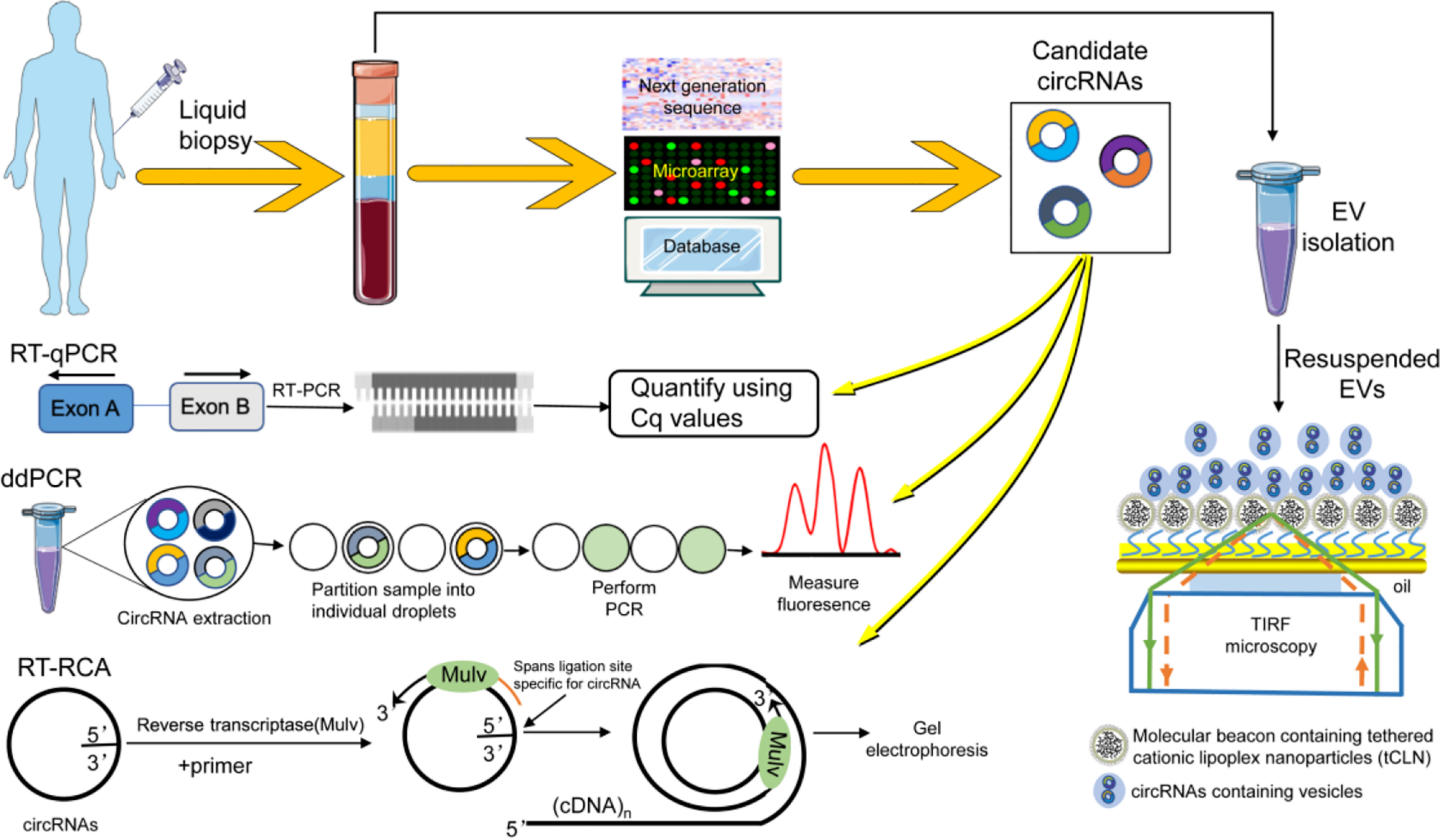
The detection method of circRNA in liquid biopsy. RNA-seq was used to identify and analyze candidate circRNAs with differential expression. Techniques such as RT-qPCR, ddPCR, RT-PCA, and tCLN were used to detect the level of circRNA in biological fluids.

The methods suitable for detecting circRNAs must distinguish circRNA from linear RNA species and be sensitive enough to allow reliable quantification of the closed-loop structure of circRNAs. The currently available technologies for circRNA assay are far from perfect (**Figure 2**) and fail to meet modern research demands. The qRT-PCR methods the most widely used technique for circRNA analysis, but it cannot distinguish between circRNAs and linear RNAs when the linear genome is used as a template to design the primer. Microarray technology is an effective and relatively sensitive technology to test and investigate circRNAs (99), but it can test only known circRNAs. As for traditional RNA sequencing (RNA-seq), another widely used method in circRNA research, the sequence reads spanning backsplicing junction sites of circRNA are filtered out in standard bioinformatics pipelines (34). The head-to-tail junction is the only instant proof of a circRNA. The testing efficiency is relatively low as only 0.1% of RNA-seq can contain this peculiar bond, and only part of circRNAs can be detected in one specimen (21). Besides, the next-generation sequencing (NGS) has other defects in circRNA detection, especially the incomplete capture of full-length circRNAs and the difficulty in accurate circRNA quantification. Although there are some strategies making up for the shortcomings, the short sequence obtained by NGS sequencing cannot be solved fundamentally. The third-generation high-throughput single-molecule sequencing technology has great advantages in the field of circular RNA research. Its long read-length eliminates the over-fragmentation characteristics of second-generation sequencing fundamentally, which brings hope about the detection of the full length of circRNA and accurate quantification. In addition, the biggest advantage of third-generation sequencing is that it can completely capture RNA transcript molecules. Unlike the NGS, which requires algorithms to splice, this allows us to see the complete sequence of the transcript rather than guessing by the algorithm. This ability guarantees the natural advantage of studying variable shear events. However, third-generation sequencing is inherently inadequate in the accuracy of single-base sequencing. Therefore, if single-base resolution studies are to be conducted, such as gene mutations, it is often necessary to perform multiple repetitive detections on the same location or combine with the second-generation sequencing for correction. The latest research strategies of third-generation sequencing technology in the circRNA field are isoCirc (101) and CIRI-long (102). They have optimized the detection technology based on conventional third-generation sequencing technology, which not only ensures the detection of the full-length circRNA detection, reduced low-accuracy defects, high detection rate, low expression levels of circRNA detection, and the advantages of mitochondrial circRNAs detection, thereby guaranteeing the future of circRNA research.

Compared with the traditional circRNA detection methods, reverse transcription rolling circle amplification (RT-RCA) assay has the advantages of simple operation, low cost, and high sensitivity (103). It can even distinguish the circRNA from the linear RNA bearing the same sequence, which is superior to most methods such as qRT-PCR. Droplet digital polymerase chain reaction (ddPCR) is another novel nucleic acid testing method that dilutes template DNA into non-interacting droplets (104). However, these two methods also have shortcomings in large-scale multiplex detection of genes. Additionally, these technologies, such as qRT-PCR, microarray, and NGS, use RNA isolation procedures to mix the RNA released by the tumor cells with the circRNA from all other non-tumor cells. Therefore, these technologies only measure the total amount of circRNA in body fluids and cannot detect the circRNA from specific cells or vesica. Their clinical application is also limited because of their cumbersome and time-consuming procedures and high costs.

Cationic liposome nanoparticle (tCLN) biochips are advanced over the previous technologies. The positively charged chips trap the negatively charged exosomes in solution by attaching tCLN coated with a specific molecular probe to the chip. The molecular probes in nanoparticles can recognize specific substrates in exosomes and generate corresponding fluorescence signals. This method is simple and cost-effective, has good repeatability, and can effectively detect the differences between different exosomes compared to the currently available methods. Although it is mainly used to detect the microRNA (miRNA) or messenger RNA (mRNA) content in vesicles to predict cancer (105, 106), the technology can also be used for circRNA detection. However, this technique can only perform a qualitative RNA expression analysis and not perform a precise quantitative analysis. Moreover, a single circRNA test can predict cancers with a certain probability of missed detection. In the future, it may be necessary to use several circRNAs as a platform to jointly detect their expression and make diagnoses and predictions based on the comprehensive expression. In the light of insufficient detection methods discussed so far, further developing detection methods, development is required before an ideal circRNA detection method is found.

## The Potential Application of CircRNAs in Anticancer Therapy

After years of clinical and basic research, RNA-based cancer therapy is booming, and various oligonucleotides have been approved for clinical trials, the most successful of which is the cancer therapy based on interfering RNA (siRNA) or antisense oligonucleotide (ASO). Earlier research data have confirmed that the siRNA can treat various solid tumors, including liver cancer, pancreatic cancer, lung cancer, prostate cancer, breast cancer, and ovarian cancer. Among them, two lipid carrier-based siRNA drugs ALN-VSP02 and TKM-080301 targeting the vascular endothelial growth and Polo-like kinase 1 (*PLK1*) gene for liver cancer treatment have completed phase I clinical trials (1). The polymer carrier-based siRNA implant siG12D LODER for treating pancreatic cancer has entered phase II clinical trials. Additionally, the nucleic acid drug Trabedersen (OT-101) based on ASO has been approved for the treatment of brain tumors treatment and is currently undergoing phase III clinical trials (107, 108). Although there is no preclinical report on using circRNAs alone as a therapeutic target, the strategy for targeting circRNAs is emerging rapidly from the laboratories.

**Figure 3:**
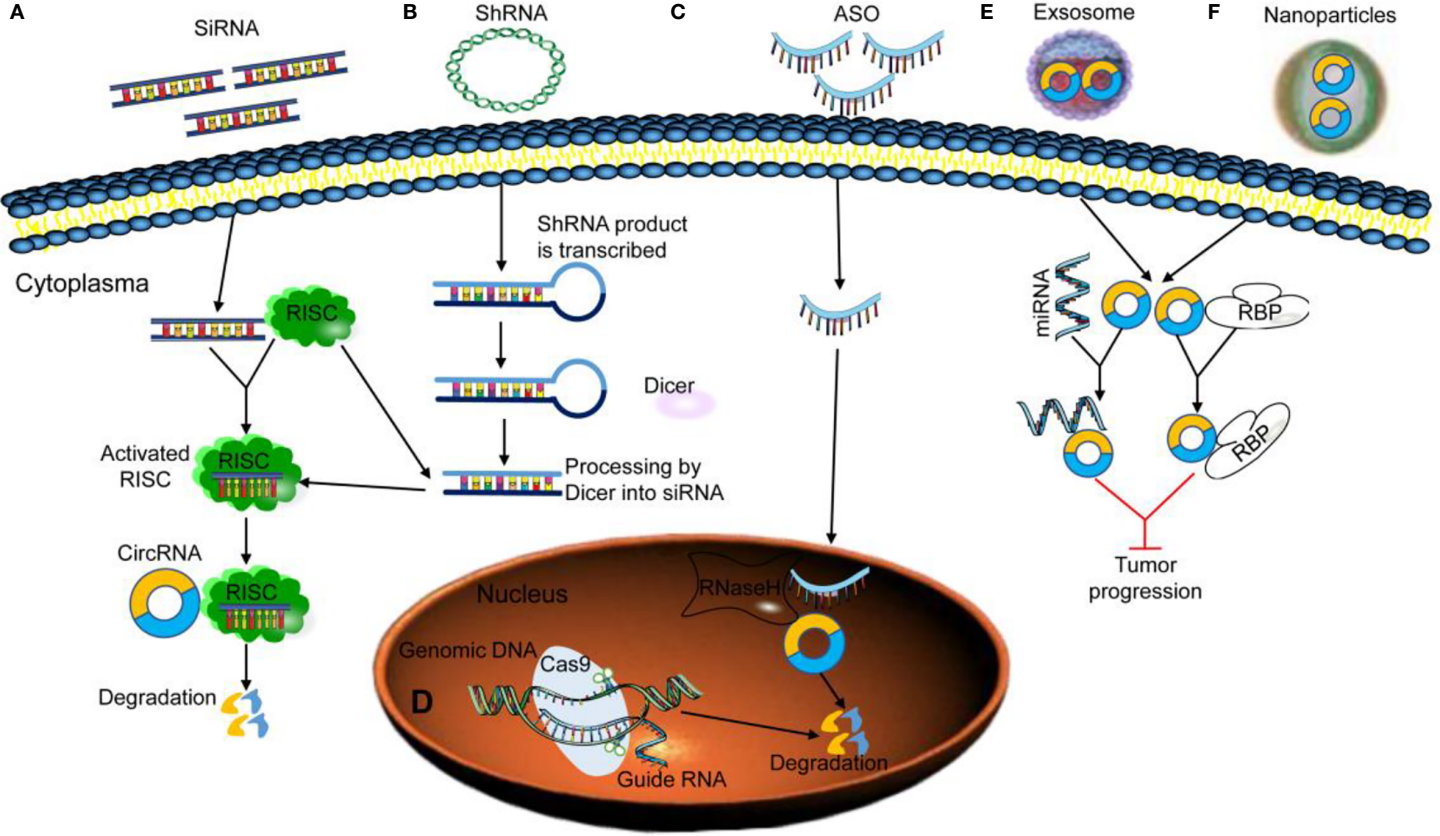
Strategies for targeting circRNAs. **(A, B)** Exogenous siRNA/shRNA activates RNA-induced silencing complex (RISC) and interacts with its specific target circRNA to induce degradation. **(C)** Exogenous antisense oligodeoxynucleotide (ASO) activates RNase H to induce circRNA to degradation. **(D)** Knockout of circRNA-associated genomic DNA using clustered regularly interspaced short palindromic repeats (CRISPR)/Cas9. **(E, F)** The loss of specific circRNAs can be rescued by reintroducing exosomal or nanoparticles’ circRNA into the cells as miRNA or RNA-binding protein (RBP) sponges to inhibit tumor progression.

Recently, several approaches have been developed to study circRNAs and target circRNAs for therapeutic purposes of cancer *in vivo*. The primary strategy is to intervene in cancer carcinogenesis, metastasis, apoptosis, and drug resistance by knocking down or overexpressing circRNAs, including circRNA knockdown strategies mediated by siRNA, short hairpin RNA (shRNA), ASO, and CRISPR/cas9 (109) (**Figures 3A** **–D**), the circRNA overexpression strategies mediated by vector and synthesis of circRNAs (110, 111), and circRNA delivery strategies mediated by exosome or nanoparticle (**Figures 3** **E, F**) (112, 113). Numerous studies have revealed that using siRNAs to target degradation or knock down the expression of circRNAs *in vivo* or *in vitro* is the most commonly used strategy for cancer therapy with circRNAs as the therapeutic target (114). Zhang et al. reported the successful knockdown of circNRIP1 by cholesterol-conjugated circNRIP1 siRNA in patient-derived xenograft (PDX) mouse models and reduced the PDX tumor growth *via* downstream metabolism alterations mediated by the AKT/mTOR signaling pathway (73). Wang et al. also demonstrated that silencing circHIPK3 through intratumorally injected si-circHIPK3 exhibits an inhibitory effect on CRC growth and metastasis in xenograft animal models (115). However, the siRNA-mediated circRNA knockdown strategies might have some obstacles, such as rapid degradation, delivery barriers, off-target effects, and immunogenicity. ASOs, DNA, or RNA molecules with a length of 8–50 nt can be an ideal alternative for siRNAs to knock down the circRNA expression by targeting intron sequences or reversing splice junctions and pre-mRNAs (116) (**Figure 3C**). Moreover, some ASOs that target circRNAs are also complementary to the intron-containing pre-mRNA; it is possible that these ASOs bind directly to pre-mRNA, leading to its degradation and causing reduced mRNA and circRNA expression. Therefore, careful selection of ASOs’ target sequence is necessary to avoid unwanted degradation of the parental RNA.

CRISPR/Cas9 systems have lower mismatch tolerance and inhibit circRNAs more specifically than RNAi (**Figure 3D**). Rajewsky et al. (117) confirmed that a circRNA CircHIPK3, usually upregulated in liver cancer, could be targeted and inhibited by specially designed gRNAs of CRISPR/Cas9 *in vitro*; in subsequent mouse models, another circRNAs Cdr1as could be selectively knocked out by the CRISPR/Cas9 strategy. The success of this experiment confirmed the feasibility of CRISPR/Cas9 technology for circRNAs *in vivo*. However, the CRISPR-Cas9-mediated knockout may affect the parental mRNA expression. Recent research showed that some Cas9 enzyme variants could mediate RNA-dependent RNA degradation in a programmable and site-specific manner (118). The technology could be helpful to directly target circRNAs at their unique splice junction, thereby circumventing the non-specificity against the parental mRNA while providing better selectivity and safety. These studies have indicated that the CRISPR/Cas9 system may be one of the most promising therapeutic strategies for clinical cancer treatment with circRNAs as the therapeutic target.

Reintroducing circRNAs as a drug into cells and inhibiting the invasion, metastasis, drug resistance, and inducing apoptosis of cancer cells by overexpressing certain important circRNAs is another strategy for cancer treatment. Using a natural circRNA molecule to treat cancer has the advantage of mimicking the endogenous physiological situation. However, targeted delivery of circRNAs with specific functions to human cells is still difficult, especially for safe delivery *in vivo* (119). Commonly used circRNA overexpression strategies include plasmid vector delivery and viral vector delivery. The plasmid vector construction process is relatively simple; however, its application is restricted due to low transfection efficiency. Compared with plasmid vectors, the viral vectors have obvious advantages. One can achieve efficient and stable transduction of different cells and animals using different viral vector tools. However, the circRNA expression viral vectors could also generate unexpected side effects. Penaud-Budloo et al. observed that despite significant improvements in upstream and downstream processes, adeno-associated virus (AAV) stocks still contain significant impurities that should be minimized during production; otherwise, systemic administration of very high doses may result in immunotoxicity (120). Similarly, the strategy of vector delivery to achieve the circRNA overexpression should also confront the non-specific and potentially harmful effects caused by the by-products of wrong splicing. Highly purified circRNA molecules synthesized *in vitro* may be used to overcome these shortcomings. However, the artificially synthesized circRNAs are immunogenic, which may be due to the lack of the m6A modification of exogenous circRNAs. Additionally, exosomes can be used as stable natural transport carriers to deliver specific circular RNAs through tumor cell adsorption (**Figure 3E**). Chen et al. (121) found that exosomal circ-0051443 decreases the tumor growth through competitive binding to miR-331-3p and promotes BAK1 upregulation *in vivo* and *in vitro*. Zhang et al. (122) also found that exosomal CircGDI2 suppresses oral squamous cell carcinoma progression through the MiR-424-5p/SCAI axis regulation. Thus, exosomes may transport specific circRNAs to target tissue and play a prominent role in cancer intervention treatment.

Various studies have confirmed nanoparticles as good drug carriers that make significant application progress in the delivery of various clinical drugs (**Figure 3F**) (123). Zhao et al. found that nanoparticles loaded with circRNA SCAR successfully delivered circRNA SCAR to the mitochondria of hepatocyte cells *in vivo*, improving the glucose tolerance and insulin resistance of mice on a high-fat diet and alleviated nonalcoholic steatohepatitis (NASH) effectively (124). Additionally, Wu et al. (125) successfully used the circ-Foxo3 plasmid-PEG-gold nanoparticle complex to complete the delivery of circRNA to cancer cells. They also verified the effect of circ-RNAFoxo3 in inhibiting the tumor growth *in vivo*. The new gold nanoparticle carrier has a good potential for circular RNA delivery, although its biological safety remains to be studied.

Many obstacles still remain in the field of cancer treatment targeting circRNAs. However, in the coming years, with a better understanding of the functional mechanism of circRNAs and further development of methods targeting the specificity and effectiveness of circRNAs *in vivo*, the clinical potential of circRNA-based cancer treatment will be further explored. The broad selection (cancer or genetically well-defined rare diseases) and good therapeutic effects of RNA-targeted therapy have attracted widespread interest from academic research institutions and pharmaceutical companies (Orna Therapeutics); increased revenues from approved RNA therapies have led to increased investments in this field (126). Further research will only make liquid biopsy and therapeutic strategy with circRNAs an important tumor diagnosis and treatment method.

## Challenges and Perspectives

Although circRNAs in body fluids are closely associated with cancer, their application as a great source of biomarkers for cancer diagnosis deserves further prospective verification. Biofluids are latent cancer biomarkers; nevertheless, there is still a wide gap between the discovery and clinical validation of biomarkers and also the clinical enforcement of these trials. Therefore, several hurdles must be overcome before circRNA biomarkers can be translated into clinical applications. One of the major concerns is that the methods for discovering and analyzing circRNAs are far from ideal, although enormous progress has been made in the past decades (127, 128). Future research need to gauge the analytical performance of disparate circRNA analysis in clinical specimens, such as RNA-seq, circRNA microarray, RT-qPCR, ddPCR, and tCLN (103, 128). Attention must be paid to standardizing in circRNA discovery and analysis when estimating the sensitivity and specificity (129). It must be noted that the circRNA biomarkers of biofluids in these studies (**Table 1**) represent only elementary features of biomarkers in cancers. The methods used in most of these studies are case–control studies on small samples with apparent phenotypes. Additional clinical specimens are needed to verify their sensitivity and specificity in a larger cohort, especially by identifying patients with similar clinical symptoms and translating them into clinical availability. Furthermore, their utility in illness diagnosis or prognostic prediction also requires detection in a prospective study. The greatest challenge in translating circRNA research from bench to bedside lies in the lack of a potentially designed and independently verified biomarker research. Further research is required to detect and verify the biomarkers’ availability in clinical medicine, such as their power to improve outcomes. The rapid and accurate detection of circRNAs from different tissues or fluids is also a significant obstacle to promote circRNAs in clinical liquid biopsy. Finally, linking the circRNA expression to other molecular changes obtained in cancer is also the goal of the follow-up research. Previous research has found that circRNA expression, KRAS, and EGFR mutations are associated with lung and colorectal cancer (130, 131). However, most published research has not studied this potential correlation. CircRNA-based cancer treatment strategies also face challenges about the choice of treatment vectors and treatment effects. In short, the circRNA research remains in its primary stages, and its effect on tumorigenesis has only begun to be illuminated. The discoveries till now make circRNA a prospective cancer biomarker and a potential target in cancer therapy. Although there are multiple challenges and questions to be faced and answered, the prospective possibility of translating the biofluid circRNA biomarkers into clinical applications gives us novel and hopeful options for liquid biopsy.

## Data Availability Statement

The original contributions presented in the study are included in the article/supplementary material. Further inquiries can be directed to the corresponding author.

## Author Contributions

YZ and YW conducted the literature review and drafted the manuscript. WL, XS, and PW revised the manuscript. All authors have read and agreed to the published version of the manuscript.

## Funding

This work was supported by grants from the National Key R&D Program of China (2018YFC2000400) and the National Natural Science Foundation of China (81772270).

## Conflict of Interest

The authors declare that the research was conducted in the absence of any commercial or financial relationships that could be construed as a potential conflict of interest.

## Publisher’s Note

All claims expressed in this article are solely those of the authors and do not necessarily represent those of their affiliated organizations, or those of the publisher, the editors and the reviewers. Any product that may be evaluated in this article, or claim that may be made by its manufacturer, is not guaranteed or endorsed by the publisher.
